# Genetic variation of *ABCB1* (rs1128503, rs1045642) and *CYP2E1* rs3813867 with the duration of tuberculosis therapy: a pilot study among tuberculosis patients in Indonesia

**DOI:** 10.1186/s13104-021-05711-8

**Published:** 2021-07-31

**Authors:** Melisa Intan Barliana, Arif Satria Wira Kusuma, Widya Norma Insani, Sofa Dewi Alfian, Ajeng Diantini, Mutakin Mutakin, Tina Rostinawati, Herlambang Herlambang, Irma Melyani Puspitasari, Auliya Abdurrohim Suwantika, Rizky Abdulah

**Affiliations:** 1 Department of Biological Pharmacy, Faculty of Pharmacy, Universitas Padjadjaran, Jl. Raya Bandung Sumedang KM 21, Jatinangor, Bandung, 45363 Indonesia; 2 Center of Excellence in Higher Education for Pharmaceutical Care Innovation, Universitas Padjadjaran, Jatinangor, Bandung, Indonesia; 3 Department of Pharmacology and Clinical Pharmacy, Faculty of Pharmacy, Universitas Padjadjaran, Bandung, Indonesia; 4 Department of Pharmaceutical Analysis and Medicinal Chemistry, Faculty of Pharmacy, Universitas Padjadjaran, Bandung, Indonesia; 5 Faculty of Medicine, Jambi University, Jambi, Indonesia

**Keywords:** *ABCB1* C1236T, *ABCB1* C3435T, *CYP2E1*−1293G>C, Single nucleotide polymorphism, Risk factor

## Abstract

**Objective:**

The risk of contracting tuberculosis (TB) and the efficacy of TB therapy are affected by several factors, including genetic variation among populations. In the Indonesian population, data on the genes involved in drug transport and metabolism of TB therapy are limited. The aim of this study was to identify the genetic profile of the *ABCB1* gene (rs1128503 and rs1045642) and *CYP2E1* gene (rs3813867) in Indonesians with TB. This study was a cross-sectional study of 50 TB outpatients in Jambi city, Indonesia. Sociodemographic characteristics were obtained from medical records. Whole blood was collected, and genomic DNA was isolated. Single nucleotide polymorphisms were determined using polymerase chain reaction-restriction fragment length polymorphism with *Hae*III, *Mbo*I, and *Pst*I for rs1128503, rs1045642 (*ABCB1*), and rs3813867 (*CYP2E1*), respectively.

**Result:**

The frequency of alleles of each gene was analyzed by Hardy–Weinberg equilibrium. The genetic profiles of *ABCB1* rs1128503 and rs1045642 were varied (CC, CT, TT), while *CYP2E1* rs3813867 was present in CC (wild type). The genetic variations of *ABCB1* and *CYP2E1* may have no significant correlation with the duration of TB therapy. Nevertheless, this study may provide as preliminary results for the genetic profiles of *ABCB1* (rs1128503, rs1045642) and *CYP2E1* (rs3813867) in the Indonesia population.

**Supplementary Information:**

The online version contains supplementary material available at 10.1186/s13104-021-05711-8.

## Introduction

Tuberculosis (TB) is an infectious disease with a high mortality rate. TB is caused by *Mycobacterium tuberculosis* (*M. tuberculosis*) that mainly invades the lung. Indonesia has a pulmonary TB prevalence of 0.42% (1,017,290 cases), and in 2018, the incidence rate was 316 cases per 100,000 population, making the country third highest in the world for TB [1, 2]. Moreover, TB resistant to rifampicin or multiple drug resistant tuberculosis (MDR-TB) is a serious threat. In 2018, there were 24,000 cases of MDR-TB in Indonesia [2].

First-line TB therapy is an adequate strategy for TB-sensitive cases; a combination of rifampicin, isoniazid, pyrazinamide, and ethambutol (2-month intensive phase) is continued with rifampicin and isoniazid for 4 months. These regimens are strongly recommended for countries with a high incidence of MDR-TB [3–5]. Strategies of TB eradication focus on monitoring therapy and comprehensive patient care; however, the efficacy of therapy still presents challenges, especially in developing countries [6, 7]. Current TB therapy achieves > 95% cure and < 5% relapse rates, but a small proportion of patients are not responsive to the therapy [8]. Factors affecting the efficacy of TB therapy include the host and bacteria. Genetic variations among individuals are known to affect the efficacy and toxicity of therapy [9]. Single nucleotide polymorphisms (SNPs) of genes involved in the metabolism or uptake of TB drugs show correlations with efficacy, such as the cytochrome P-450 (CYP) family and adenosine triphosphate (ATP)-binding cassette (ABC) family [10–12]. Alterations in these genes may influence the pharmacokinetics, sensitivity, or adverse reactions to drugs [13].

Adenosine triphosphate (ATP)-binding cassette B1 (ABCB1) is a membrane transporter, which is encoded by the *ABCB1* gene, plays an important role in ATP-dependent uptake and efflux of extracellular compounds and xenobiotics into and from cells [10, 14, 15]. Therefore, polymorphisms of *ABCB1* determine the risk factor, efficacy, and toxicity of some therapies. *ABCB1* gene C1236T (rs1128503) and/or C3435T (rs1045642) are involved in diseases such as TB, cancer, minor ischemic stroke, chronic liver disease, and mental health [14, 16–24]. The adverse effect of TB therapy is significantly correlated with hepatotoxicity [25–27]. Cytochrome P-450 2E1 (CYP2E1) encoded by the *CYP2E1* gene, is mainly expressed in the liver, and catalyze xenobiotic metabolism. CYP2E1 is involved in isoniazid metabolism, and its activity and expression are affected by polymorphisms of *CYP2E1* gene in the 5ʹ upstream region (−1053C>T) [9, 28–32].

It is important to understand whether genetic variation is one of the risk factors for TB and severity progression. The present study was the first study to identify the genetic profile of the *ABCB1* gene (rs1128503 and rs1045642) and *CYP2E1* gene (rs3813867) of TB patients in Jambi city, Indonesia.

## Main text

### Methods

#### Subjects

This study was a cross-sectional study of TB outpatients in Abdul Manap Hospital, Jambi city, Indonesia. Fifty patients were examined to observe the SNPs of *ABCB1* (rs1128503 and rs1045642) and *CYP2E1* (rs3813867). The sample collection was conducted for 3 months. The population (male and female) that met the inclusion criteria, such as patients diagnosed with TB and currently undergoing TB therapy, was selected. The sociodemographic characteristics (age, gender, education, and occupation), duration of therapy, therapy category, smoking status, and alcohol consumption were obtained from medical records.

#### Genotyping of *ABCB1* (rs1128503 and rs1045642) and *CYP2E1* (rs3813867)

The gene sequences were obtained from The National Center for Biotechnology Information (https://www.ncbi.nlm.nih.gov). Whole blood was collected and genomic DNA was isolated using *Purelink Genomic DNA Mini Kit* (Thermo Fischer Scientific, Waltham, MA, USA). SNP identification was determined using the polymerase chain reaction-restriction fragment length polymorphism (PCR-RFLP) method. PCR was conducted by *PCR SuperMix* (Thermo Fisher Scientific, Waltham, MA, USA) using specific primers for each SNP. All specific primer (Invitrogen, Thermo Fisher Scientific, Waltham, MA, USA) sequences were obtained from previous studies [18, 33]. Each specific primer was confirmed with GENETYX version 10 software and Oligo Calc: Oligonucleotide Properties Calculator (http://www.basic.northwestern.edu/biotools/oligocalc.html) (Additional file 1: Table S1). PCR products were digested with *Mbo*I, *Hae*III, or *Pst*I (Promega, Madison, WI, USA), electrophoresed using 2.5% agarose gel containing SYBR^®^ Safe DNA Gel Stain (Thermo Fischer Scientific, Waltham, MA, USA), and visualized under ultraviolet light at 312 nm. The *Mbo*I-digested PCR fragment (*ABCB1* rs1045642) produced 88- and 162-bp for CC (wild-type); 88-, 162-, and 250-bp for CT; 250-bp for TT. *Hae*III digested PCR fragments (*ABCB1* rs1128503) produced 270-, 65-, and 35-bp for CC (wild-type); 270-, 100-, 65-, and 35-bp for CT; 270- and 100-bp for TT. The *Pst*I digested PCR fragment (*CYP2E1* rs3813867) produced: 412-bp for CC (wild-type), 118-, 294-, and 412-bp for CT, and 294-, and 412-bp for TT. *GAPDH* gene expression was used as an internal PCR control in the same samples.

#### Statistical analysis

Each locus was analyzed for allele frequencies using descriptive statistics with Hardy–Weinberg equilibrium (HWE, *df* = 1). Statistically significant differences in sociodemographic characteristics were analyzed using Student’s *t*-test with *p* < 0.05 considered as statistically significant. The correlation between sociodemographic characteristic and duration of therapy was analyzed by Chi-square or Fisher exact test univariate analysis.

### Results

#### Sociodemographics of TB patients

TB patients in Jambi city were mostly males of productive age (40 years old of age or younger) (Table 1; Additional file 2: Figure S1 and Additional file 3: Figure S2). Most had graduated from senior high school and were either housewives or industrial laborers. Smoking and alcohol consumption status were only observed in only 16% and 6% of patients, respectively.

**Table 1:** Correlation of sociodemographic characteristics with duration of therapy (n = 50)

Risk factors	n (%)	Duration of therapy ≤ 6 months n (%)	Duration of therapy > 6 months n (%)	*p* value
Gender				1.000^*^
Male	35 (70.0)	26 (68.4)	9 (75.0)	
Female	15 (30.0)	12 (31.6)	3 (25.0)	
Age group, years (N)				–
≤ 29	9 (18.0)	9 (23.7)	0 (0)	
1	8 (16.0)	6 (15.8)	2 (16.7)	
40–49	13 (26.0)	11 (28.9)	2 (16.7)	
50–59	12 (24.0)	7 (18.4)	5 (41.7)	
≥ 60	8 (16.0)	5 (13.2)	3 (25.0)	
Education (N)				–
Primary school	4 (8.0)	3 (7.9)	1 (8.3)	
Junior high school	7 (14.0)	5 (13.2)	2 (16.7)	
Senior high school	37 (74.0)	28 (73.7)	9 (75.0)	
Diploma/bachelor degree	2 (4.0)	2 (5.3)	0 (0)	
Occupation (N)				–
Students/university students	4 (8.0)	4 (10.5)	0 (0)	
Employed	27 (54.0)	21 (55.3)	6 (50.0)	
Unemployed	19 (38.0)	13 (34.3)	6 (50.0)	
Smoking status (N)				0.125^#^
No	34 (68.0)	28 (73.7)	6 (50.0)	
Yes	16 (32.0)	10 (26.3)	6 (50.0)	
Alcohol consumption				0.621^*^
No	44 (88.0)	34 (89.5)	10 (83.3)	
Yes	6 (12.0)	4 (10.5)	2 (16.7)	
Genotype *ABCB1* rs1045642				0.668^*^
CC	9 (18.0)	6 (15.8)	3 (25.0)	
CT + TT	41 (82.0)	32 (84.2)	9 (75.0)	
Genotype *ABCB1* rs1128503				0.386^*^
CC	8 (16.0)	7 (30.4)	1 (11.1)	
CT + TT	25 (50.0)	16 (69.6)	8 (88.9)	
Missing	17 (34.0)	–	–	
Genotype *CYP2E1* rs3813867				–
CC	50 (100)	12 (24)	38 (76)	–
CT + TT	0 (0)	–	–	

Statistical comparisons were performed using ^#^Chi-square test and ^*^Fisher exact test

#### Genotyping of *ABCB1* (rs1128503 and rs1045642) and *CYP2E1* (rs3813867)

**Fig. 1:**
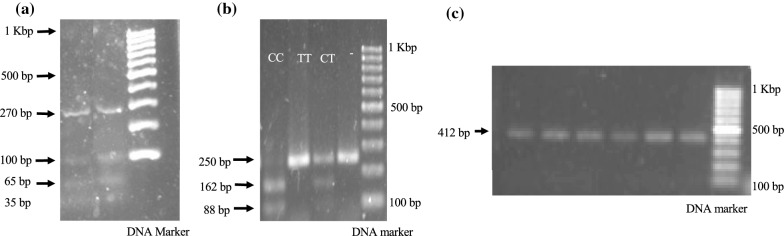
Visualization of **a** *ABCB1* rs1128503, **b** *ABCB1* rs1045642, and **c** CYP2E1 rs3813867 using agarose gel electrophoresis

All samples were clearly identified by the RFLP method, but 17 samples were not identified for *ABCB1* rs1128503 (RFLP by *Hae*III) (Table 1, Fig. 1). Results showed that the genetic profiles of *ABCB1* rs1128503 were 7 (14%) CC, 14 (28%) CT, 12 (24%) TT, and 17 (34%) were not identified; *ABCB1* rs1045642 was 9 (18%) CC, 25 (50%) CT, and 16 (32%) TT. The T allele of both SNPs in *ABCB1* showed higher frequency than the C allele (Table 1). The *CYP2E1* rs3813867 was all observed in the CC (wild type) genotype (Additional file 4: Figure S3). Otherwise, the allele frequency of each genotype of all SNPs was not significant in the disequilibrium state, with *p* > 0.05 according to the Hardy–Weinberg equation (Additional file 5: Table S2; Additional file 6: Table S3; Additional file 7: Table S4).

#### Duration of therapy and genetic variation

Most of patients were in first line therapy for TB (< 6 months of therapy), but 1 patient was in the extensive phase, and 4 patients were confirmed to have MDR-TB (> 6 months of therapy) (Table 1). All of the sociodemographic determinants were not significantly correlated with the duration of therapy (*p* > 0.05). The genotype of *ABCB1* (rs1045642 or rs1128503) showed variations rather than continuing for correlation analysis with duration of therapy. The variations in *ABCB1* were divided into the C allele (CC, wild-type) and T allele (CT, TT) but showed no significant correlation with the duration of TB therapy (Table 1).

### Discussion

Sociodemography characteristics are considered as risk factors for TB and MDR-TB incidence [34]. Our results showed that TB cases were higher in men, similar to most studies in Malaysia [35]. Active TB cases are shown to affect individuals of productive age, as shown in a Java island, Indonesia study [35–38]. However, we observed 17 patients who were less than 40 years of age and 33 who were more than 40 years old. Jambi city is located on Sumatera Island and might have different sociodemographic characteristics, including various ethnicities, from other parts of Indonesia.

Educational background is one TB determinants. A lower level of education correlated with a higher TB infection rate [35, 39]. Similarly, our data showed that 48 out of 50 patients had at least a senior high school educational. Lower educational background was assumed to have less exposure to health information; however this assumption has not been proven since TB-related health information can also be accessed by those with informal education [35, 40]. In the present study, most TB patients were employed. In Malaysia, employment status as a determinant of TB infection was showed that patients who were unemployed completed TB therapy at higher rates [35]. Our result was similar to a study in Kenya [41]. Productive age and employed status of individuals are risk factors for TB infection because of high mobility and the increased likelihood of being exposed to TB [35, 42]. Behavioral factors, such as smoking status, alcohol consumption, and drug abuse, made individuals more susceptible to TB infection, thus affecting the incidence of active TB [43, 44]. We observed that only 16 patients smoked and 6 patients consumed alcohol. This might indicate that other factors affect the incidence of TB in Indonesia, especially in Jambi Province. Because the majority of Indonesians are Muslim, alcohol consumption is prohibited. Due to data limitations, we cannot statistically calculate the correlation of such sociodemographic characteristics with TB infection.

ABCB1 is involved in diseases and the efficacy of therapy, where SNPs in the *ABCB1* gene affect its function. Polymorphisms of *ABCB1* rs1128503 and rs1045642 are the most studied variants genetic of *ABCB1* in diseases and indicate high frequencies in several populations [45, 46]. This was the first genotype variation study of *ABCB1* in Indonesian TB patients, especially in the Jambi population, although we did not find a significant association with sociodemography characteristics or duration of therapy. We did find a higher number of T alleles in both polymorphisms of *ABCB1*. T alleles of *ABCB1* rs1128503 are major alleles in Asia and minor alleles in Africa [14]. Both polymorphisms of *ABCB1* rs1045642 and rs1128503 are synonymous SNPs, but they alter the stability of mRNA expression; therefore, they affect the drug pharmacokinetics, whether through reduced or increased drug bioavailability. An *ABCB1* genotype study in Brazil showed that SNP rs1128503 had a significant correlation with the risk for MDR-TB. One of the limitations of our study was that we did not correlate the genetic variation with clinical outcomes or efficacy of therapy due to a lack of data. However, this study may become a preliminary study to identify the genetic profile of *ABCB1* rs1128503 (C1236T) and rs1045642 (C3435T) in Indonesia, especially in Jambi Province.

In the present study, all samples had the wild-type (CC) genotype; therefore we could not analyze the correlation with sociodemographic characteristics and duration of therapy. The variation in the *CYP2E1* rs3813867 genotype in Malaysia (Asian and non-Asian) and in Turkey showed similar results to our study [47, 48]. The activity of CYP2E1 was isoniazid level-dependent and involved in acetyl hydrazine oxidation into diacetyl hydrazine and acetyl diazene ketene which was hepatotoxic [49]. *CYP2E1* variation affects the efficacy of therapy, especially in adverse events causing anti-TB drug-induced hepatotoxicity (ATDH) [32]. Wild-type of *CYP2E1* rs3813867 (c1/c1) was found to have increased activity compared to other variants of genotypes [9, 49]. These differences in the clinical outcomes of *CYP2E1* rs3813867 have been studied in several populations. In Turkey, the heterozygosity of *CYP2E*1 rs3813867 was observed to increase the risk of ATDH, while in the North Indian population, the wild-type had a lower risk [25, 48]. As in China, most of Uyghur genetic variations were c1/c1 but not significantly associated with ATDH [50]. In our study, we found correlation between the genetic variation and ATDH. However, it is a potential issue for further investigation.

## Conclusions

The *ABCB1* and *CYP2E1* genetic variations may have no significant correlation (*p* > 0.05) with the duration of TB therapy, although variations was occurred in *ABCB1*, due to small sample size. The result of the present work may provide as preliminary data on *ABCB1* (rs1128503, rs1045642) and *CYP2E1* (rs3813867) genetic profiles in Indonesian populations.

## Limitations

- *ABCB1* (rs1128503, rs1045642) and *CYP2E1* (rs3813867) were considered responsible for drug efflux or metabolism, but due to the small number of patients, we could not find the significant involvement of these SNPs.
- We did not use a positive control for RFLP, although we used the GAPDH primer as an internal control for PCR.
- The visualization of PCR fragments was low quality due to low of sample concentrations; however, specific bands of correct size were observed.

## Supplementary Information

**Additional file 1: Table S1.** Primers for SNP genotyping [18, 33].

**Additional file 2: Figure S1.** Gender of TB patients.

**Additional file 3: Figure S2.** Age range of TB patients.

**Additional file 4: Figure S3.** Genotype distribution of a) *ABCB1* rs1128503, b) *ABCB1* rs1045642, and c) *CYP2E1* rs3813867.

**Additional file 5: Table S2.** Hardy–Weinberg Equilibrium for the Genotype of *ABCB1 *rs1128503.

**Additional file 6: Table S3.** Hardy–Weinberg Equilibrium for the Genotype of *ABCB1 *rs1045642.

**Additional file 7: Table S4.** Hardy–Weinberg Equilibrium for the Genotype of *CYP2E1* rs3813867.

## Data Availability

All data generated or analysed during this study are included in this published article and available in the www.figshare.com repository [https://figshare.com/articles/dataset/Genotype_of_Samples_for_ABCB1_and_CYP2E1_pdf/14680884].

## Abbreviations

ABCB1: Adenosine triphosphate (ATP)-binding cassette B1
ATDH: Anti-TB drug-induced hepatotoxicity
CYP2E1: Cytochrome P-450 2E1
HWE: Hardy–Weinberg equilibrium
MDR-TB: Multiple drug resistant tuberculosis
*M. tuberculosis*: *Mycobacterium tuberculosis*
SNPs: Single nucleotide polymorphisms
PCR-RFLP: Polymerase chain reaction-restriction fragment length polymorphism
TB: Tuberculosis
