# Supplementary material for: Genetic variation of ABCB1 (rs1128503, rs1045642) and CYP2E1 rs3813867 with the duration of tuberculosis therapy: a pilot study among tuberculosis patients in Indonesia
Source: BMC Res Notes. 2021 Jul 31;14:295. doi: 10.1186/s13104-021-05711-8 (PMC8325820; doi:10.1186/s13104-021-05711-8)
Supplement: Supplementary file 1 — Additional file 1: Table S1. Primers for SNP genotyping [18, 33]. [file 13104_2021_5711_MOESM1_ESM.docx]

Table S1. Primers for SNP genotyping [18, 33]

| Primer | | Sequence |
| --- | --- | --- |
| *ABCB1* rs1128503 | F | 5’TATCCTGTGTCTGTGAATTGCC’3 |
|  | R | 5’CCTGACTCACCACACCAATG’3 |
| *ABCB1* rs1045642 | F | 5’TGTTTTCAGCTGCTTGATGGC3’ |
|  | R | 5’TGCTCCCAGGCTGTTTATTTGA3’ |
| *CYP2E1* rs3813867 | F | 5'-CCAGTCGAGTCTACATTGTCA-3' |
|  | R | 5 -TTCATTCTGTCTTCTAACTGG-3' |
