# Supplementary figures and images for: Genetic variation of ABCB1 (rs1128503, rs1045642) and CYP2E1 rs3813867 with the duration of tuberculosis therapy: a pilot study among tuberculosis patients in Indonesia
Source: BMC Res Notes. 2021 Jul 31;14:295. doi: 10.1186/s13104-021-05711-8 (PMC8325820; doi:10.1186/s13104-021-05711-8)

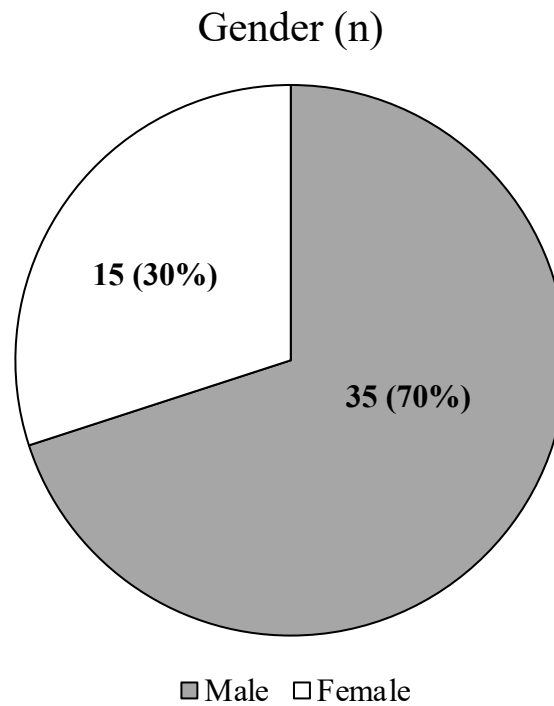

Figure S1. Gender of TB patients

Supplement: Supplementary file 2 — Additional file 2: Figure S1. Gender of TB patients. [file 13104_2021_5711_MOESM2_ESM.pdf]

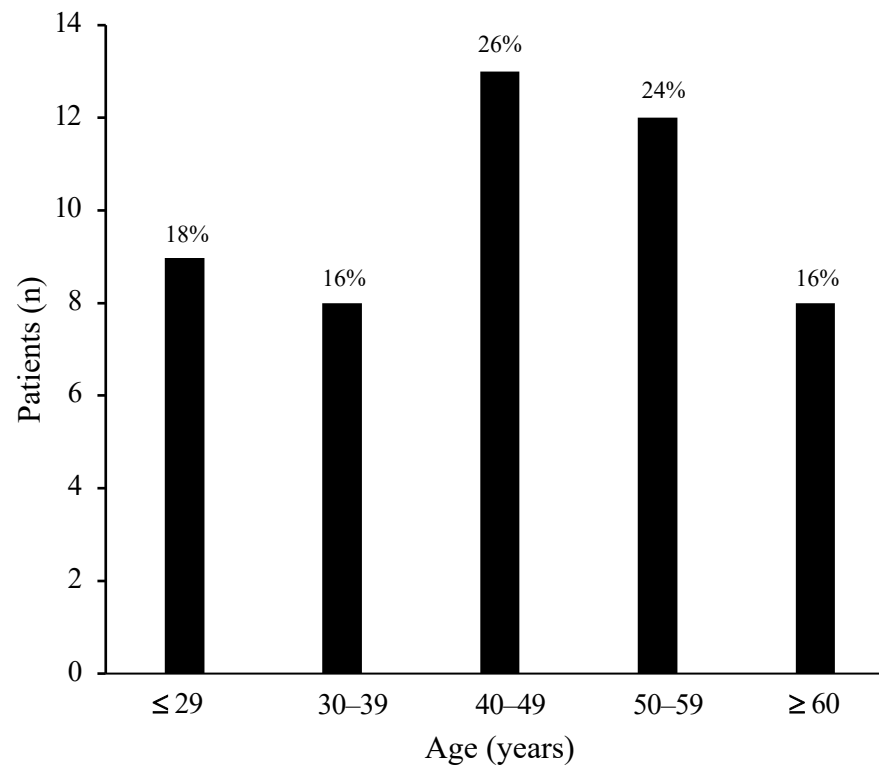

Figure S2. Age range of TB patients

Supplement: Supplementary file 3 — Additional file 3: Figure S2. Age range of TB patients. [file 13104_2021_5711_MOESM3_ESM.pdf]

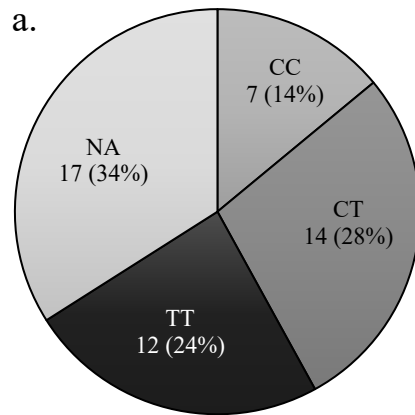

ABCB1 rs1128503

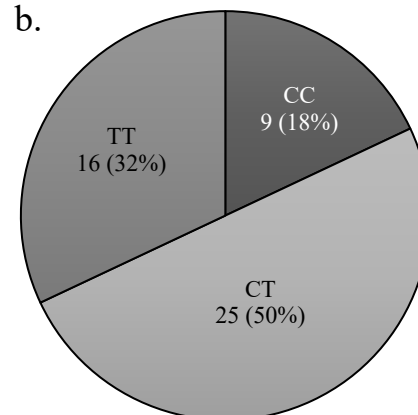

ABCB1 rs1045642

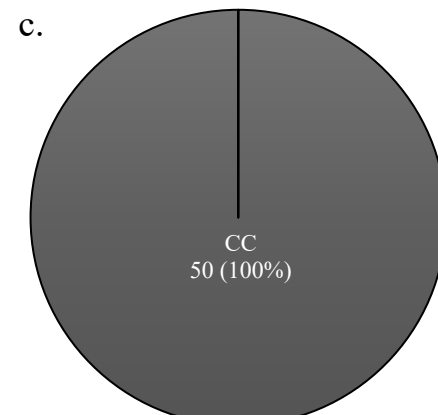

CYP2E1 rs3813867

Figure S3. Genotype distribution of a) *ABCB1* rs1128503, b) *ABCB1* rs1045642, and c) *CYP2E1* rs3813867

Supplement: Supplementary file 4 — Additional file 4: Figure S3. Genotype distribution of a) ABCB1 rs1128503, b) ABCB1 rs1045642, and c) CYP2E1 rs3813867. [file 13104_2021_5711_MOESM4_ESM.pdf]
