# Supplementary material for: Genetic variation of ABCB1 (rs1128503, rs1045642) and CYP2E1 rs3813867 with the duration of tuberculosis therapy: a pilot study among tuberculosis patients in Indonesia
Source: BMC Res Notes. 2021 Jul 31;14:295. doi: 10.1186/s13104-021-05711-8 (PMC8325820; doi:10.1186/s13104-021-05711-8)
Supplement: Supplementary file 5 — Additional file 5: Table S2. Hardy–Weinberg Equilibrium for the Genotype of ABCB1 rs1128503. [file 13104_2021_5711_MOESM5_ESM.docx]

Table S2. Hardy-Weinberg Equilibrium for the Genotype of *ABCB1* rs1128503

| Genotype | Observation | | Expectation | |
| --- | --- | --- | --- | --- |
| *wild type* (CC) | | 7 | 5.9 |  |
| Heterozygote (CT) | | 14 | 16.1 |  |
| Homozygote mutant (TT) | | 12 | 10.9 |  |
| Variation of allele frequency | 0.58 | |  | |
| X^2^ value | 0.57 | |  | |
| *p-value* | 0.449 | |  | |
