# Supplementary material for: Genetic variation of ABCB1 (rs1128503, rs1045642) and CYP2E1 rs3813867 with the duration of tuberculosis therapy: a pilot study among tuberculosis patients in Indonesia
Source: BMC Res Notes. 2021 Jul 31;14:295. doi: 10.1186/s13104-021-05711-8 (PMC8325820; doi:10.1186/s13104-021-05711-8)
Supplement: Supplementary file 6 — Additional file 6: Table S3. Hardy–Weinberg Equilibrium for the Genotype of ABCB1 rs1045642. [file 13104_2021_5711_MOESM6_ESM.docx]

Table S3. Hardy-Weinberg Equilibrium for the Genotype of *ABCB1* rs1045642

| Genotype | Observation | Expectation |
| --- | --- | --- |
| *wild type* (CC) | 9 | 9.2 |
| Heterozygote (CT) | 25 | 24.5 |
| Homozygote mutant (TT) | 16 | 16.2 |
| Variation of allele frequency | 0.57 |  |
| X^2^ value | 0.019 |  |
| *p-value* | 0.886 |  |
